# Whole-Genome Sequence of Sungri/96 Vaccine Strain of Peste des Petits Ruminants Virus

**DOI:** 10.1128/genomeA.00056-14

**Published:** 2014-02-13

**Authors:** Manjunath Siddappa, Ravi Kumar Gandham, Vishal Sarsani, Bishnu Prasad Mishra, Bina Mishra, C. G. Joshi, A. P. Sahoo, A. K. Tiwari, Sarath Chandra Janga

**Affiliations:** a Division of Veterinary Biotechnology, Indian Veterinary Research Institute, Izatnagar, Uttar Pradesh, India; b Department of Biohealth Informatics, School of Informatics and Computing, Indiana University Purdue University, Walker Plaza Building, Indianapolis, Indiana, USA; c Department of Animal Biotechnology, College of Veterinary Science and Animal Husbandry, Anand Agricultural University, Anand, Gujarat, India

## Abstract

We report the complete genome sequence of the Sungri/96 vaccine strain of peste des petits ruminants virus (PPRV). The whole-genome nucleotide sequence has 89 to 99% identity with the available PPRV genome sequences in the NCBI database. This study helps to understand the epidemiological and molecular characteristics of the Sungri/96 strain.

## GENOME ANNOUNCEMENT

Peste des petits ruminants virus (PPRV) is a negative-sense, single-stranded RNA virus belonging to the genus *Morbillivirus* of the family *Paramyxoviridae*. It causes an acute viral disease, peste des petits ruminants (PPR), in sheep and goats, with high mortality rates of 90 to 100% observed in naive populations (1). The full genome of PPRV has 15,948 bp encoding six structural proteins in a 3′ to 5′ direction (3′-N–P-M-F–H-L-5′) (2). Four genetically distinct lineages of the virus (I to IV) have been defined all over the world based on molecular epidemiology of the F and N gene sequences of the virus (3). Lineages I to III are prevalent in Africa and other countries, and lineage IV is found to be prevalent in India (4, 5).

The Sungri/96 PPRV vaccine that is widely used in India provides long-term immunity in both sheep and goats (4). In the present study, the whole genome of this Sungri/96 strain was assembled from RNA sequencing data generated from goat peripheral blood mononuclear cells (PBMCs) infected with PPRV Sungri/96. Total RNA was isolated from infected PBMCs and sequenced on an Ion Torrent platform. The quality reads (mean Phred score, >25), processed with prinseq-lite.pl, were aligned with the available PPRV reference genome from NCBI (accession no. NC_006383) using Bowtie 2.0 (6). The aligned reads were assembled using MacVector 12.7.5 (2012, MacVector, Inc.).

The genome sequence of Sungri/96 shows 96 to 99% identity with the Asian isolates and 89 to 92% identity with the African isolates. Phylogenetic analysis of the complete genome sequences revealed that the Sungri/96 vaccine strain clusters with the Asian isolates to a common node away from the African isolates. Among the available full-genome sequences, the Nigerian isolates (accession no. EU267274, X74443, and HQ197753), the Côte d’Ivoire isolate (accession no. EU267273), and the Asian isolates (accession no. AJ849636, JF939201, FJ905304, and JX217850) were classified under lineages I, III, and IV, respectively (3). The clustering of the Sungri/96 isolate with the Asian isolates to a common node indicates that it belongs to lineage IV, as confirmed earlier in a comparison of partial gene sequences (3).

This report on the complete genome sequence of the Sungri/96 vaccine strain will help to better understand the epidemiological and molecular characteristics of the Sungri/96 strain.

### Nucleotide sequence accession number.

The whole genome sequence was deposited in the GenBank under the accession no. KF727981.
